# Tailoring the crystallographic orientation and thickness of indium sulfide thin films for enhanced photoelectrochemical water splitting

**DOI:** 10.1039/d5lf00219b

**Published:** 2025-09-03

**Authors:** Xiuru Yang, Arthur Graf, Hong Chang, Yongde Xia, Asif Ali Tahir, Yanqiu Zhu

**Affiliations:** a Faculty of Environment, Science and Economy, University of Exeter Exeter EX4 4QF UK y.zhu@exeter.ac.uk; b HarwellXPS, Research Complex at Harwell, Rutherford Appleton Lab Didcot OX11 0FA UK; c School of Chemistry, Cardiff University Main Building, Park Place Cardiff CF10 3AT UK; d Solar Energy Research Group, Environment and Sustainability Institute, Faculty of Environment, Science and Economy, University of Exeter Penryn Campus Penryn TR10 9FE UK

## Abstract

Indium sulfide thin films play a crucial role in photoelectrochemical (PEC) water splitting, offering promising strategies to mitigate energy shortages and global warming. In this study, indium sulfide thin films were synthesized *via* a hydrothermal method, and the effects of sulfur precursors—l-cysteine (LC) and l-cysteine hydrochloride (LCHCl)—along with hydrothermal temperature ramp rates (3 and 10 °C min^−1^) on their crystallographic orientation, morphology, and thickness were investigated. The findings revealed that films synthesized with LC predominantly exhibited the (440) facet, while those synthesized with LCHCl had the (311) facet. Additionally, films produced at 3 °C min^−1^ were thicker than those synthesized at 10 °C min^−1^. The film (LC-IS-10) synthesized at 10 °C min^−1^ using LC achieved a photocurrent density of 3.7 mA cm^−2^ at −0.2 V *vs.* Ag/AgCl, which outperformed that of LCHCl-synthesized film (LCHCl-IS-10) at the same heating rate (2.6 mA cm^−2^) and those of the films synthesized at 3 °C min^−1^ (LC-IS-3: 0.7 mA cm^−2^ and LCHCl-IS-3: 2 mA cm^−2^). 2-Hour photocurrent stability assessments indicated that LC-synthesized films (LC-IS-3: 1 mA cm^−2^, LC-IS-10: 670 μA cm^−2^) exhibited superior stability to the LCHCl-synthesized films (LCHCl-IS-10: 90 μA cm^−2^, LCHCl-IS-3: 33 μA cm^−2^). This improved stability was attributed to their (440) facet, which was structurally more compact and symmetric and exhibited reduced sulfur exposure than the less ordered (311) facet. Although the LC-IS-3 film had the lowest photocurrent density, it showed enhanced stability, owing to the thickness alteration caused by the oxidation of surface S^2−^ species. This research provides insights for optimizing material design in PEC water-splitting applications, advancing sustainable energy solutions.

## Abstract 2

Hydrothermally synthesized indium sulfide thin films using l-cysteine and l-cysteine hydrochloride exhibit distinct crystallographic orientations, morphologies, thicknesses, and PEC water splitting performances.

## Graphical abstract

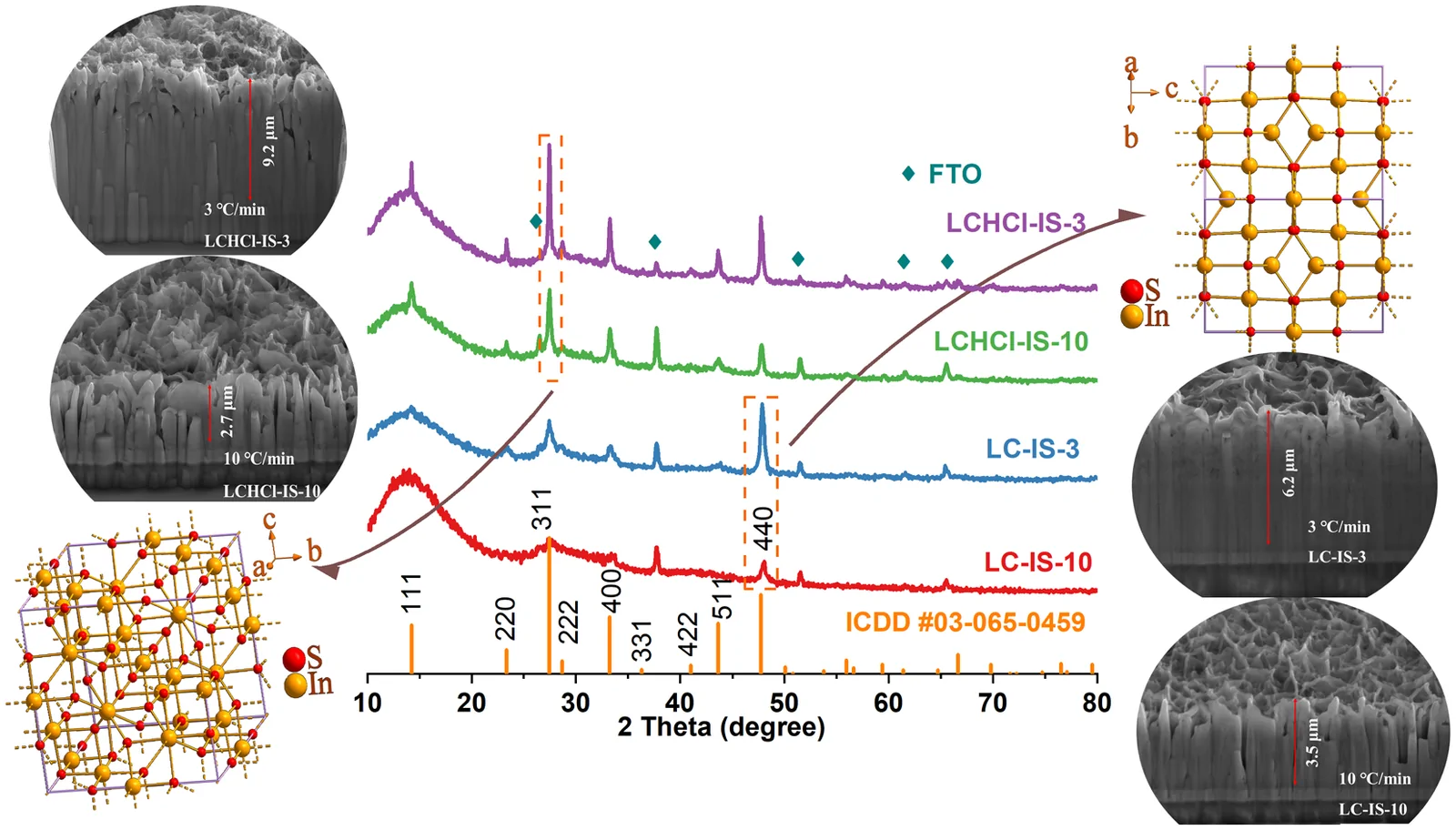

## Introduction

According to the Statistical Review of World Energy (2024, 73rd edition),^1^ the current energy system remains heavily dependent on non-renewable energy resources—oil, natural gas, and coal—which account for over 80% of global primary energy consumption. The BP Statistical Review of World Energy (June 2014) projected that these resources would be exhausted in approximately 53, 55, and 113 years, respectively.^2,3^ This ongoing reliance on these non-renewable energy sources to power industrial and urban activities has posed a significant challenge to limiting the global average surface temperature rise to 1.5 °C.^4^ The resulting increase in temperature has exacerbated extreme weather events, including large-scale wildfires, devastating floods, intense heatwaves, tornadoes, droughts, and severe storms.^5^ To mitigate these issues and the imminent energy shortage, the transition to a more sustainable and renewable energy system is needed. Hydrogen, which has the highest energy density by weight (33.3 kWh kg^−1^), produces no carbon emission, and is derived from abundant hydrogen resources, plays a critical role in advancing this transition.^6^ The reported renewable methods for hydrogen evolution include electrolysis, bio-hydrogen production, photocatalysis, thermochemical cycles, plasmolysis, and photoelectrochemical (PEC) water splitting.^7^ Among these, PEC water splitting stands out due to its direct use of sunlight, lower energy requirements, simplified system design, environmental benefits, and potential for higher efficiency. However, the widespread application of PEC water splitting is constrained by the limited efficiency, short-term stability, high costs, and scalability issues of advanced semiconductor photoelectrodes (both photocathodes and photoanodes).

Continuous efforts are being made to design suitable photoelectrodes, examples involving numerous metal sulfide thin films, including In_2_S_3_,^8^ ZnS,^9^ CdS,^10^ Cu_2_S,^10^ MoS_2_,^11^ SnS_2_,^12^ ZnIn_2_S_4_,^13^ CdIn_2_S_4_,^14^ CuInS_2_,^15^ being explored for PEC water splitting. These materials are favoured due to their narrow band gaps, which enable them to absorb a large portion of solar light compared with many other metal oxides.^16^ Among them, In_2_S_3_ thin films are notable for their optimal band gap (2.0–2.3 eV), high light absorption coefficient, non-toxicity, and superb photoelectric conversion efficiency.^17–19^ Considerable research has been conducted to synthesize high-quality In_2_S_3_ thin films, and the selection of sulfur precursors and indium complexes played a pivotal role in achieving the desired film properties. For instance, Cao's group utilized acetic acid as a ligand to form a complex with In^3+^ and thioacetamide as the sulfur source. By systematically varying the amount of acetic acid in the chemical bath deposition process, they were able to produce In_2_S_3_ thin films with varying thicknesses and achieved an optimal photocurrent density of 41.93 μA cm^−2^*vs.* Ag/AgCl under a 100 mW cm^−2^ (AM 1.5 G) light source in a 0.5 M Na_2_SO_4_ electrolyte.^20^ Similarly, Ehsan's group highlighted the significance of indium complexes in controlling the film morphology. They synthesized four distinct indium complexes: [In(S_2_CNCy_2_)_3_]·2py, [In(S_2_CN(iPr)_2_)_3_]·1.5py, [In(S_2_CPip)_3_]·0.5py, and [In(S_2_CNBzMe)_3_], which were used as precursor for deposition onto fluoride-doped tin oxide (FTO) substrates *via* aerosol-assisted chemical vapour deposition (AACVD).^21^ These complexes were important for forming β-In_2_S_3_ thin films with distinct morphologies, leading to a photocurrent density of 1.25 mA cm^−2^ at 0.23 V *vs.* Ag/AgCl.^21^ Furthermore, the combination of indium–thiourea and indium–diethylene glycol (DEG) complexes underscored the complementary roles of sulfur precursor and indium complexes in the synthesis process. DEG regulated the concentration of In^3+^ ions in the solution, while thiourea provided the necessary OH^−^ and S^2−^ ions that facilitated the formation of flower-like porous structures.^22^ The resulting In_2_S_3_ thin film demonstrated a transient photocurrent density of 1.3 mA cm^−2^*vs.* 0.2 V *vs.* RHE.^22^

l-Cysteine (LC), a naturally occurring sulfur-containing amino acid, offers a more sustainable and environmentally friendly alternative to traditional sulfur sources such as thioacetamide and thiourea, which can be toxic and harmful to the environment. With its functional groups—amino (–NH_2_), carboxyl (–COOH), and thiol (–SH)—LC exhibits versatile coordination chemistry, forming stable complexes with various metal ions, including Cu^+^/Cu^2+^, Zn^2+^, Ni^2+^, Fe^3+^, Co^2+^, Mn^2+^, Cr^3+^/Cr^4+^, Pt^2+^, Pd^2+^, Cd^2+^, Mg^2+^, In^3+^, and Au^3+^ through its functional groups.^23^ This makes it an ideal precursor for the synthesis of metal sulfides. Moreover, the formed metal complexes can act as stabilizing and dispersing agents, preventing agglomeration and ensuring the uniform distribution of noble metals such as Ag, Au, and Pd, as well as metal sulfides like CdS.^24–27^ This uniform distribution can enhance the catalytic performance of the host materials, particularly in applications like PEC water splitting. Additionally, LC serves as a morphology-directing reagent through the binding of its thiol group to metal ions. By controlling the concentration of LC, the morphology of the target materials can be tuned by adjusting the growth sites, the number of branches, and their dimensions.^28–30^

In this work, LC and l-cysteine hydrochloride (LCHCl) have been used as sulfur sources to synthesize indium sulfide thin films on FTO glass utilizing a hydrothermal method. We utilize two different temperature ramp rates in distinct ovens with the same sulfur source, and apply the same temperature ramp rates while varying the sulfur sources. The choice of sulfur precursors significantly affects the structural quality and properties of the resulting films. LC and LCHCl serve as both complexing agents and morphology-directing molecules, leading to variations in crystal facets and film thickness, which, in turn, directly impact the long-term PEC water splitting performance. To evaluate these effects, we systematically compare the synthesized indium sulfide thin films based on their crystal structure, composition, morphology, thickness, electronic structure, and PEC water splitting performance. This work aims to clarify how precursor selection and synthesis conditions affect the characteristics of indium thin films, highlighting their potential for PEC water splitting applications.

## Experimental section

### Materials and chemicals

Indium(iii) chloride anhydrous (InCl_3_, metals basis, 99.99%, Thermo Scientific Chemicals), l-cysteine (C_3_H_7_NO_2_S, ≥98%, Thermo Scientific Chemicals), l-cysteine hydrochloride anhydrous (C_3_H_8_ClNO_2_S, 97%, Thermo Scientific Chemicals), sodium sulfide nonahydrate (Na_2_S·9H_2_O, ≥98.0%, Sigma-Aldrich), sodium sulfite anhydrous (Na_2_SO_3_, 98%, Thermo Scientific Chemicals), and sodium sulfate anhydrous (Na_2_SO_4_, ReagentPlus®, ≥98%, Sigma Aldrich) were purchased in the UK and used without additional purification.

### Preparation of indium sulfide thin film

**Fig. 1:**
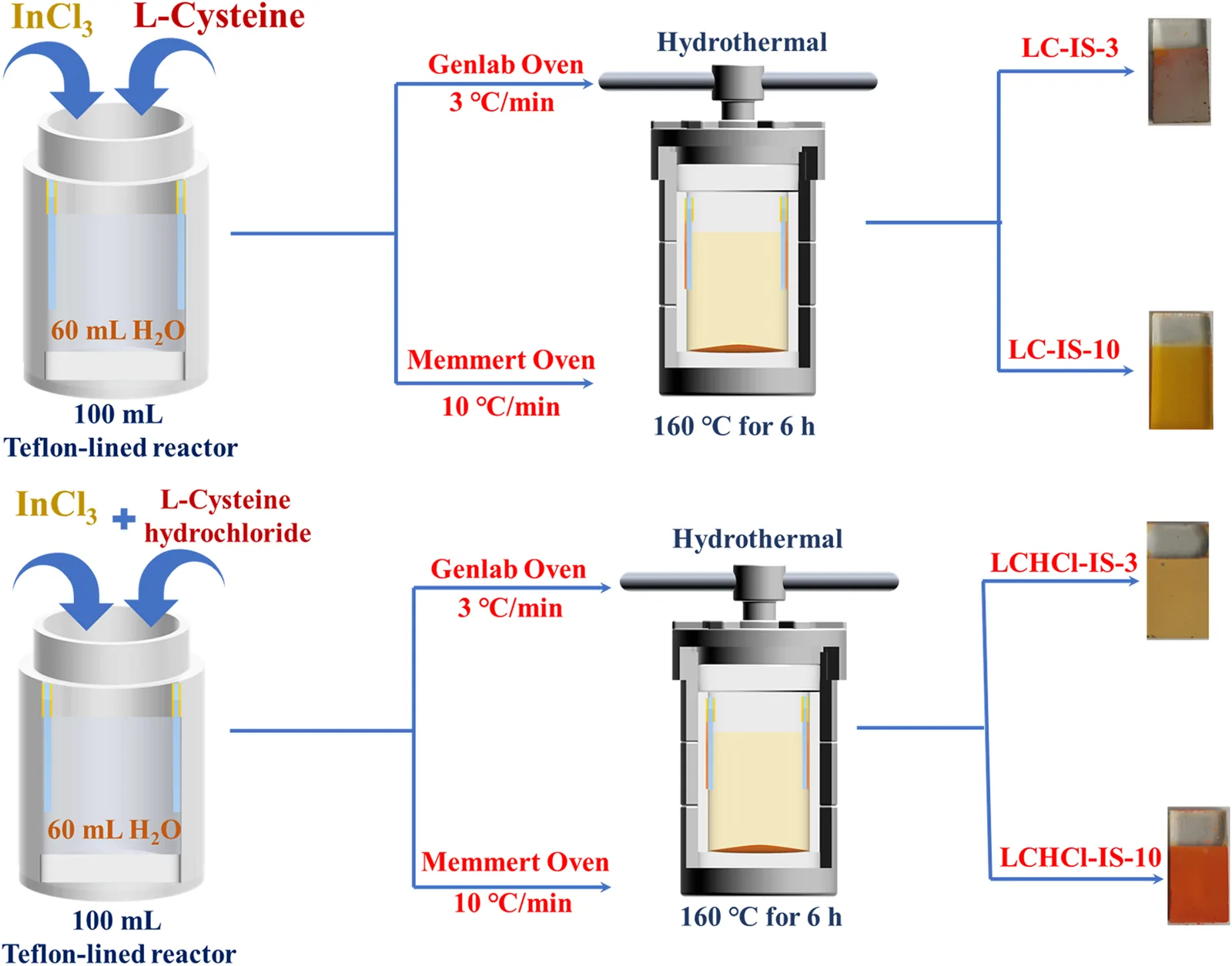
Schematic of the synthesis procedure of indium sulfide thin films.

1.5 mmol of InCl_3_, and 4.5 mmol of LC were dissolved into 60 mL of distilled water. Following 30 min of stirring, the solution was transferred into a Teflon-lined container. The top of the FTO glass was firmly wrapped with thermal tape (R-TECH Kapton, −70–250 °C) before being immersed vertically in the prepared solution, ensuring that the conductive side of the FTO glass contacted the wall of the container. The container was then moved into a stainless-steel tank and kept in an oven at 160 °C for 6 h. The resulting thin films and the precipitate collected at the bottom of the container were then washed multiple times with distilled water. Finally, the samples were dried in the oven at 80 °C for 12 h. For comparison, two ovens from different manufactures with distinct ramp rates were utilized for the thin films preparation, as the available equipment did not allow direct control of ramp rates. One oven was from Genlab, with a ramp rate of 3 °C min^−1^ in its high ramping mode, while the other, a Memmert oven, featured a ramp rate of 10 °C min^−1^. The resulting thin films were designated as LC-IS-3 and LC-IS-10, respectively. Similarly, films LCHCl-IS-3 and LCHCl-IS-10 were generated by substituting LC with an equivalent molar amount of LCHCl (Fig. 1).

### Photoelectrochemical (PEC) experiments

Under ambient air conditions, the PEC investigation was conducted using an electrochemical workstation (Metrohm Autolab (PGSTAT302N)) having a wire Pt (0.5 mm, 7.5 cm) counter electrode, an Ag/AgCl reference electrode, and a working electrode—comprising the prepared thin film. This setup utilized a mixture of 0.025 M Na_2_S·9H_2_O and 0.025 M Na_2_SO_3_ (pH = 12.32) solution as the electrolyte. A solar simulator (Newport, 1.5 AM, 300 W) was used to simulate the solar light exposure after calibrating to 1 sun using a reference silicon cell. The linear sweeping voltammetry (LSV) mode recorded the photocurrent density *vs.* applied potential curves (*J*–*V* plots) while the thin films were exposed to the chopped solar light radiation. The applied potentials (*E*_Ag/AgCl_) were transformed to the reversible hydrogen electrode (*E*_RHE_) scale using the following eqn (1).^31^

1 *E*_RHE_ = *E*_Ag/AgCl_ + 0.197 + 0.0591 × pH

Additionally, the electrochemical impedance spectroscopy (ESI) plots (Nyquist plots) were documented under the frequency response analysis (FRA) impedance potentiostatic procedure from 10 kHz to 0.1 Hz at a bias potential of −0.2 V *vs.* Ag/AgCl under the solar light irradiation. The stability of prepared thin films was assessed by analysing the photocurrent density *vs.* time plots using chronoamperometry (Δ*t* > 1 ms) mode, while exposed to the solar light irradiation at a bias of −0.2 V *vs.* Ag/AgCl.

## Characterizations

X-ray diffraction (XRD) analyses of the crystal structure for the produced thin films were conducted using a Cu Kα (*λ* = 1.54 Å) as the radiation source on a Bruker D8 Advance X-ray Diffractometer. The elemental analysis was recorded using a TESCAN VEGA3 scanning electron microscope (SEM) with an X-MAXN energy dispersive X-ray spectroscopy (EDS) detector. A focused ion beam scanning electron microscope (FIB/SEM, FEI Nova 600 Nanolab) was utilized to investigate the surface morphology of the generated thin film. A spectrometer (UV-VIS-NIR lambda 1050, PerkinElmer) was utilized to measure the ultraviolet-visible diffuse reflectance spectra (DRS) of the thin films. X-ray photoelectron spectroscopy (XPS) analysis was carried out using a Thermo NEXSA XPS system equipped with a monochromated Al Kα X-ray source (1486.7 eV), a spherical sector analyser, and three multichannel resistive plates, 128-channel delay line detectors. Measurements were performed at 19.2 W with an X-ray beam size of 400 × 200 μm. Survey scan were acquired at a pass energy of 200 eV, while high-resolution scans were recorded at a pass energy of 40 eV. Electronic charge neutralization was achieved using a dual-beam low-energy electron/ion source (Thermo Scientific FG-03) operating at an ion gun current of 150 μA and an ion gun voltage of 45 V. All data were collected at a pressure below 10^−8^ Torr and a room temperature of 294 K.

## Results and discussion

**Fig. 2:**
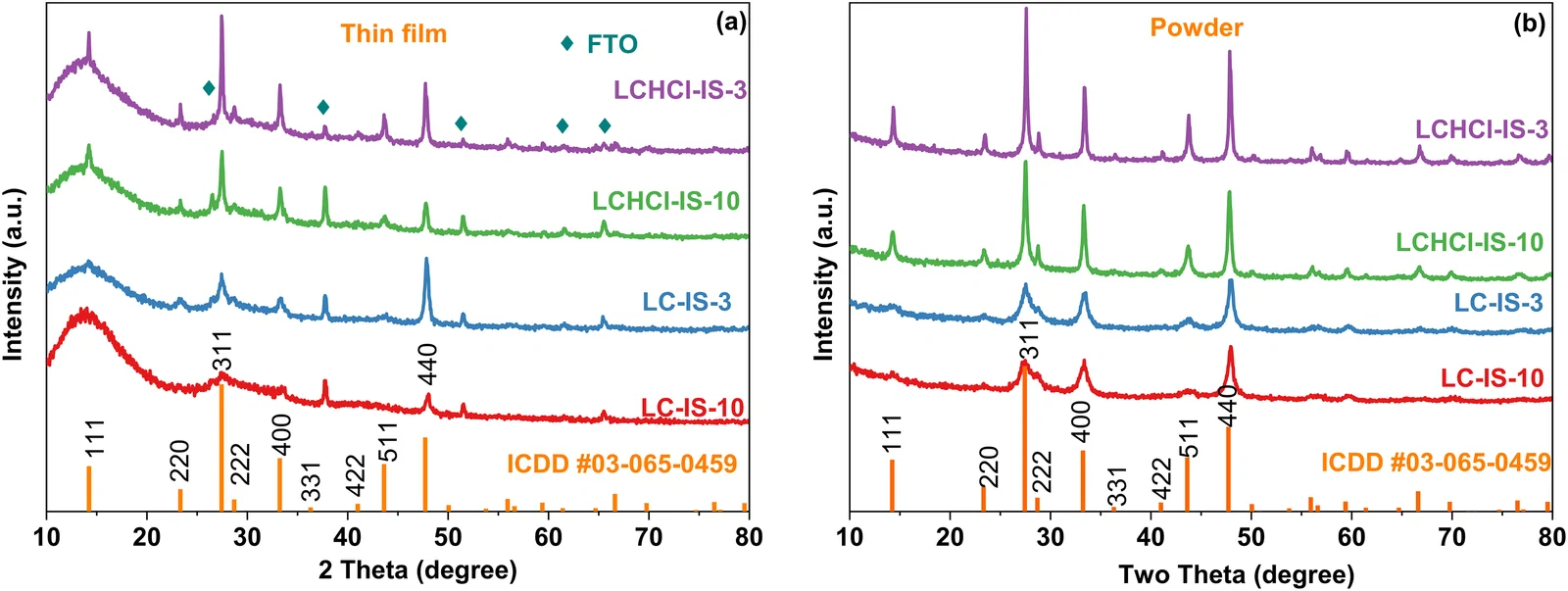
XRD patterns: (a) the as-produced indium sulfide thin films, and (b) collected corresponding powders.

The XRD results in Fig. 2 revealed the crystal structure and phase composition of the generated thin films on FTO substrates. As illustrated in Fig. 2a, all the diffraction peaks are consistent with the pure cubic indium sulfide phase, matching those in the International Centre for Diffraction Data reference (ICDD#03-065-0459).^32^ The peaks located at 2*θ* degree of 14.2, 23.3, 27.4, 28.7, 33.2, 36.3, 41.0, 43.6, and 47.7 in all thin films can be ascribed to the crystallographic planes (111), (220), (311), (222), (400), (331), (422), (511), and (440), respectively. While the peaks at 26.4°, 37.7°, 51.5°, 61.6° and 65.5° can be indexed to SnO_2_.^33^ No extra diffraction peaks were observed, indicating a relatively high purity. Furthermore, a comparative analysis of the crystallite sizes of the prepared thin films, determined using the Scherrer equation^34^ at the (440) plane, as presented in Table 1, together with the sharpness characteristics of the peaks in the XRD patterns, indicates that the LCHCl at a reduced temperature ramp rate of 3 °C min^−1^ facilitated the formation of larger crystallites. Additionally, the XRD peak intensities of the powder samples were analysed to mitigate the influence of the FTO substrate. The dominant crystallographic planes and their corresponding intensity ratios are shown in Table 2. For samples synthesized using LCHCl, LCHCl-IS-3 and LCHCl-IS-10, the (311) plane was identified as the dominant facet. In contrast, for the LC synthesized samples, LC-IS-3 and LC-IS-10, the dominant facet was the (440) plane. These variations have demonstrated that the precursor, whether LC or LCHCl, exerted a significant influence on the crystallographic orientation of the thin films.

**Table 1:** Calculated crystallite size using the Scherrer equation

Thin film	Peak position (2*θ*)	Full width at half maximum (FWHM, 2*θ*)	Crystallite size (Å)	Crystallite size (nm)
LC-IS-3	47.8	0.39	320	32
LC-IS-10	47.9	0.52	216	21.6
LCHCl-IS-3	47.7	0.25	657	65.7
LCHCl-IS-10	47.8	0.32	430	43

**Table 2:** Intensity ratios of dominant crystallographic facets from powder XRD pattern analysis

Powder	*I*_(311)_	*I*_(440)_	*I*_(311)_/*I*_(440)_
LC-IS-3	198	402	0.49
LC-IS-10	189	385	0.49
LCHCl-IS-3	1116	965	1.16
LCHCl-IS-10	851	790	1.08

The SEM-EDS spectra have been used to further identify and semi-quantitatively analyse the elemental composition of the prepared indium sulfide thin film. As shown in Fig. S1 and Table S1, when the temperature ramp rate in the hydrothermal process was 3 °C min^−1^, LC-IS-3 showed a composition of 26.1 at% indium (In) and 52.9 at% sulfur (S), maintaining a stoichiometric ratio of 1 : 2 for In to S. Meanwhile, LCHCl-IS-3 exhibited a composition of 23.6 at% In and 52 at% S, with a slightly altered ratio of 1 : 2.2 for In to S. Conversely, at a higher ramp rate of 10 °C min^−1^, both LC-IS-10 and LCHCl-IS-10 had compositions of 29.7 at% and 29.6 at% In, respectively, along with 46.7 at% and 47.1 at% S, roughly a 1 : 1.6 ratio of In to S. Notably, compared with In_2_S_3_, which has an In to S ratio of 1 : 1.5, the resulting thin films exhibited a sulfur-rich composition. The detection of Sn and O elements, originating from the FTO glass substrate, was confirmed in the thin films LC-IS-10 and LCHCl-IS-10. In contrast, Sn was not identified in the thin films LC-IS-3 and LCHCl-IS-3, which could be attributed to the increased thickness. This thickness exceeded the penetration depth of the SEM-EDS measurement, typically ranging from 1–3 μm at 20 kV. While the presence of the O and N in these two thin films (LC-IS-3 and LCHCl-IS-3) could originate from LC (or LCHCl). Moreover, N element was not detected in the thin film LC-IS-10 and LCHCl-IS-10 during the SEM-EDS analysis. However, the presence of the N 1s peaks in the XPS survey scan (Fig. S2) confirmed the existence of N in these thin films, which was likely originated from the LC/LCHCl precursors. The intensities of the N 1s peaks in the thin films synthesized at the ramp rates of 3 °C min^−1^ were higher than those of the thin films synthesized at 10 °C min^−1^. Particularly, LC-IS-3 thin films exhibited the highest intensity, possibly indicating variations in the concentration of N elements.

**Fig. 3:**
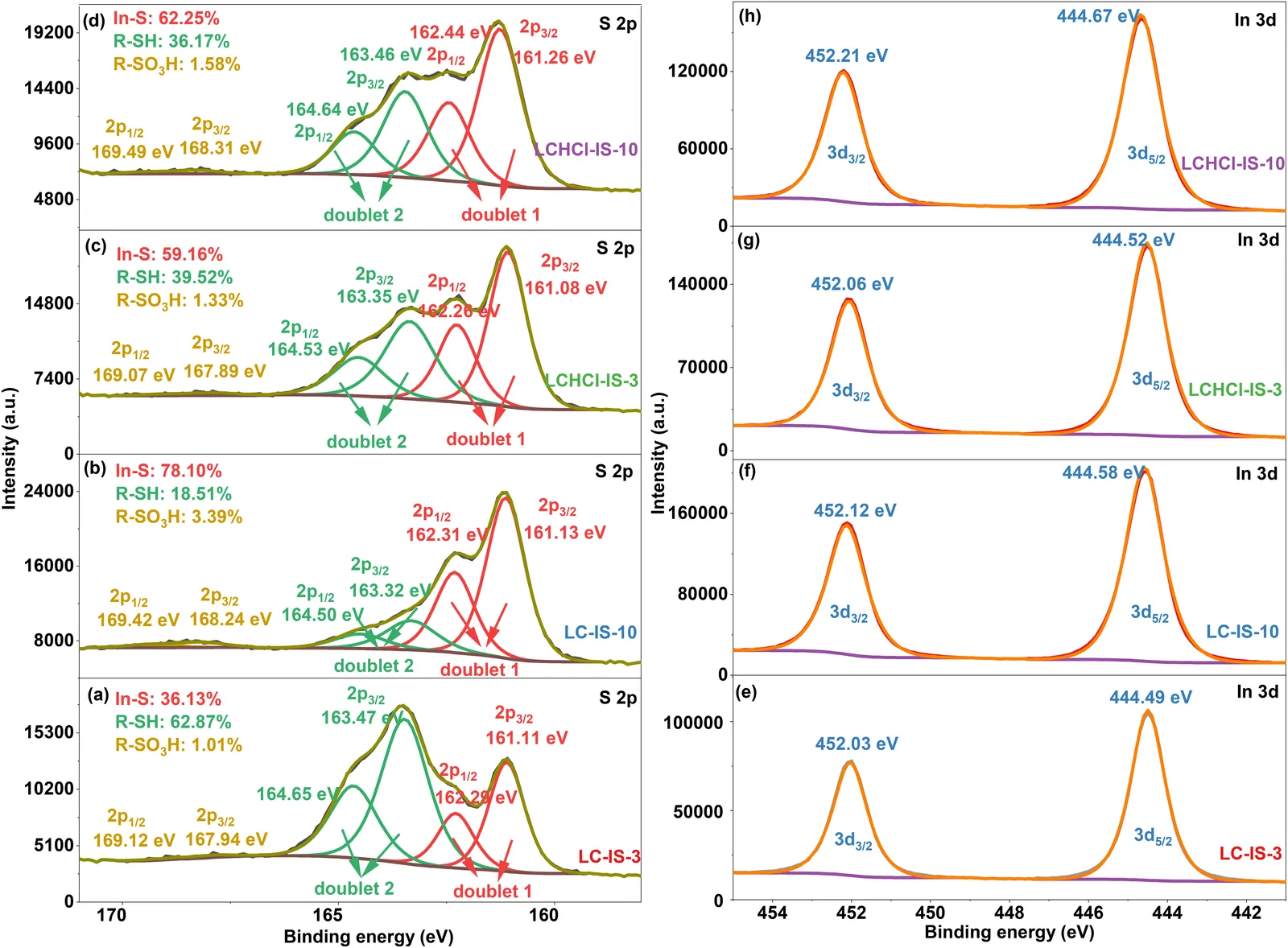
High-resolution XPS spectra of S 2p (a–d) and (e–h) In 3d.

The surface composition and chemical states of the outermost few nanometers of the synthesized thin film have been studied by performing XPS. The high-resolution S 2p XPS spectra in Fig. 3a–d can be fitted into three distinct doublets. Each doublet has two components, 2p3/2 and 2p1/2, with an area ratio of 2 : 1 attributed to the spin–orbit splitting, and a binding energy difference of 1.18 eV.^35^ Doublet 1, with S 2p3/2 at 161.11 ± 0.2 eV and S 2p1/2 at 162.29 ± 0.2 eV, corresponds to the S^2−^ species in both indium sulfide and In–cysteine complexes.^36,37^ Doublet 2, located at 163.32 ± 0.2 eV (S 2p3/2) and 164.50 ± 0.2 eV (S 2p1/2), is attributed to the C–SH functional group.^36–40^ Doublet 3, assigned to partially oxidized thiol (R–SO_3_H), observed at 167.94 ± 0.2 eV for S 2p3/2 and 169.12 ± 0.2 eV for S 2p1/2. The S 2p XPS data previously reported for LC indicated the S 2p doublet peaks corresponding to the R–SH group at 163.87 and 165.05 eV, with the C 1s binding energy at 284.8 eV used as the reference for fitting.^41^ In comparison, doublets (doublet 2) observed in all samples exhibit slight shifts to different lower binding energies across all thin films, indicating the formation and concentration variation of In–cysteine complexes.^41^

This observation is consistent with the EDS findings indicating that all thin films are sulfur-rich, suggesting that LC (or LCHCl) not only contributed to the formation of Indium sulfide but also participated in the formation of the indium(iii)–cysteine complex. Notably, as illustrated in Fig. 3a, when the hydrothermal temperature ramping rate was controlled at 3 °C min^−1^ using LC as the sulfur source, the indium(iii)–cysteine complex became the dominant component rather than indium sulfide. However, when LCHCl was used as the sulfur source, indium sulfide remained as the main component, regardless of whether the temperature ramping rate was 3 °C min^−1^ or 10 °C min^−1^. The doublet peaks of the In 3d core levels for the indium thin films are described in Fig. 3e–h. The binding energies for In 3d_5/2_ and In 3d_3/2_ are observed at 444.49 ± 0.2 eV and 452.03 ± 0.2 eV, respectively. The spin–orbit splitting between the In 3d_5/2_ and In 3d_3/2_ is 7.54 eV, consistent with the characteristic electronic state of In^3+^ within both indium sulfide and indium(iii)–cysteine complex.^38,42^ Additionally, all high-resolution C 1s XPS spectra in Fig. S3 can be deconvoluted into four distinct peaks.

The peaks observed in the range of 284.69–284.80 eV correspond to C–C from surfaces capped In–cysteine complexes.^43^ The peaks at 286.10 eV in both Fig. S3a and S3b, 286.09 eV in Fig. S3c, and 286.01 eV in Fig. S3d indicate the presence of C–O/C–N/C–S functionalities within carbon derived from LC (or LCHCl).^35,43,44^ The peaks at 287.60 eV in Fig. S3a, 287.50 eV in Fig. S3b, 287.59 eV in Fig. S3c, and 287.51 eV in Fig. S3d verify the existence of the C=O component associated with the carboxyl group structure derived from LC (or LCHCl).^35,43,44^ Moreover, peaks at 288.60 eV in Fig. S3a and b, 288.49 eV in Fig. S3c, and 288.51 eV in Fig. S3d are attributed to the carboxyl group (O=C–O), indicating the presence of carboxyl functional groups (COOH/COO^−^) originating from cysteine molecules on the film surface.^35,43–45^ In addition, the XPS spectra of all O 1s core levels (Fig. S4) can be fitted into three discernible peaks. The peak located at a higher binding energy level is attributed to the oxygen derived from COO^−^ structure, whereas the two lower-energy peaks are ascribed to the carbonyl (C=O) and hydroxyl (OH) oxygen atoms within the carboxylic acid (–COOH) structure.^35,37,41,43,44,46,47^ Thus, the XPS results have confirmed the successful synthesis of indium sulfide, with the identification of indium–cysteine complexes on the surface. The detection of these complexes has highlighted the critical role of LC and LCHCl as ligands in the formation of indium sulfide film, and in influencing its surface chemistry, which may further impact the morphological and electronic properties of the thin films.

**Fig. 4:**
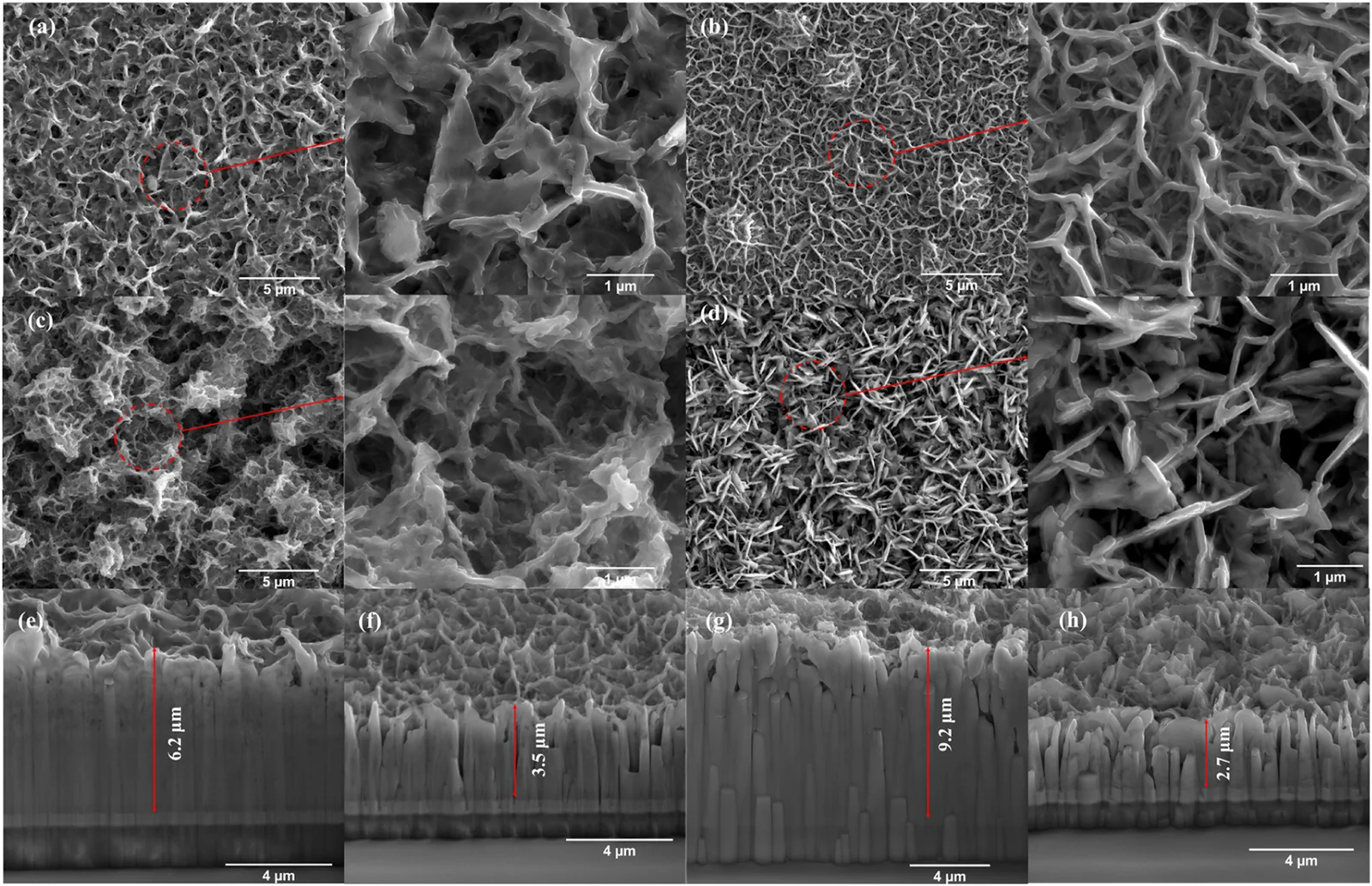
Top-view SEM image at low and high magnifications of prepared indium sulfide thin films: (a) LC-IS-3, (b) LC-IS-10, (c) LCHCl-IS-3, and (d) LCHCl-IS-10. Cross-sectional SEM images: (e) LC-IS-3, (f) LC-IS-10, (g) LCHCl-IS-3, (h) LCHCl-IS-10.

The morphology of indium sulfide thin films fabricated with different sulfur sources (LC and LCHCl) and varying temperature ramp rates in the hydrothermal process are shown in Fig. 4. As illustrated in Fig. 4a, the thin film synthesized with LC at a ramp rate of 3 °C min^−1^ exhibited uniformly distributed flake-like structure with soft and flexible edges spreading across the FTO substrate. When the temperature ramp rate was 10 °C min^−1^, the interconnected edges formed a network of pores and openings, which resembled the overlapping petals and intricate centres of marigold flowers. The key difference is that this structure grew vertically on the FTO glass rather than forming a pinhole-based microsphere. Additionally, the thin film exhibited a distinct morphology compared with that of LC-IS-3, with the edges shown in Fig. 4b being sharper and less flexible, characterized by well-defined edges and more rigid openings in the structure. When LCHCl was used as the sulfur source, the thin film at the ramp rate of 3 °C min^−1^ showed a sponge-like pattern with soft, loose, and flexible openings, as shown in Fig. 4c. However, at 10 °C min^−1^ ramp rate, the morphology of LCHCl-IS-10 sample more closely resembled a marigold flower, with a nano-petal network structure. The interconnected petal-like network structure formed a shaper and more rigid openings, as shown in Fig. 4d. The cross-section SEM images showed the variations in both thickness and morphology of the indium sulfide thin films resulting from changes in sulfur sources and the temperature ramp rates during the hydrothermal process. The cross-section SEM images also showed that the wafer stick-like structures were grew vertically on the surface of the FTO glass, exhibiting different surface morphologies as displayed in the top-view SEM images. The thickness of the indium sulfide thin film synthesized at the temperature ramp rate of 3 °C min^−1^ was 6.2 μm for LC-IS-3 and 9.2 μm for LCHCl-IS-3, which was greater than that obtained at the temperature ramp rates of 10 °C min^−1^, with 3.5 μm for LC-IS-10 and 2.7 μm for LCHCl-IS-10. Hence, the thickness of the indium sulfide thin film was significantly influenced by the temperature ramp rates, while the use of different sulfur sources greatly affected the top-view morphologies and had a slight effect on the thickness of the thin films. These variations in morphology and thickness, induced by different sulfur sources and hydrothermal temperature ramp rates, can further influence the key properties of the indium sulfide thin films, such as optical activity, charge transfer, and charge separation efficiency. These factors, in turn, may affect the performance of PEC water splitting.

**Fig. 5:**
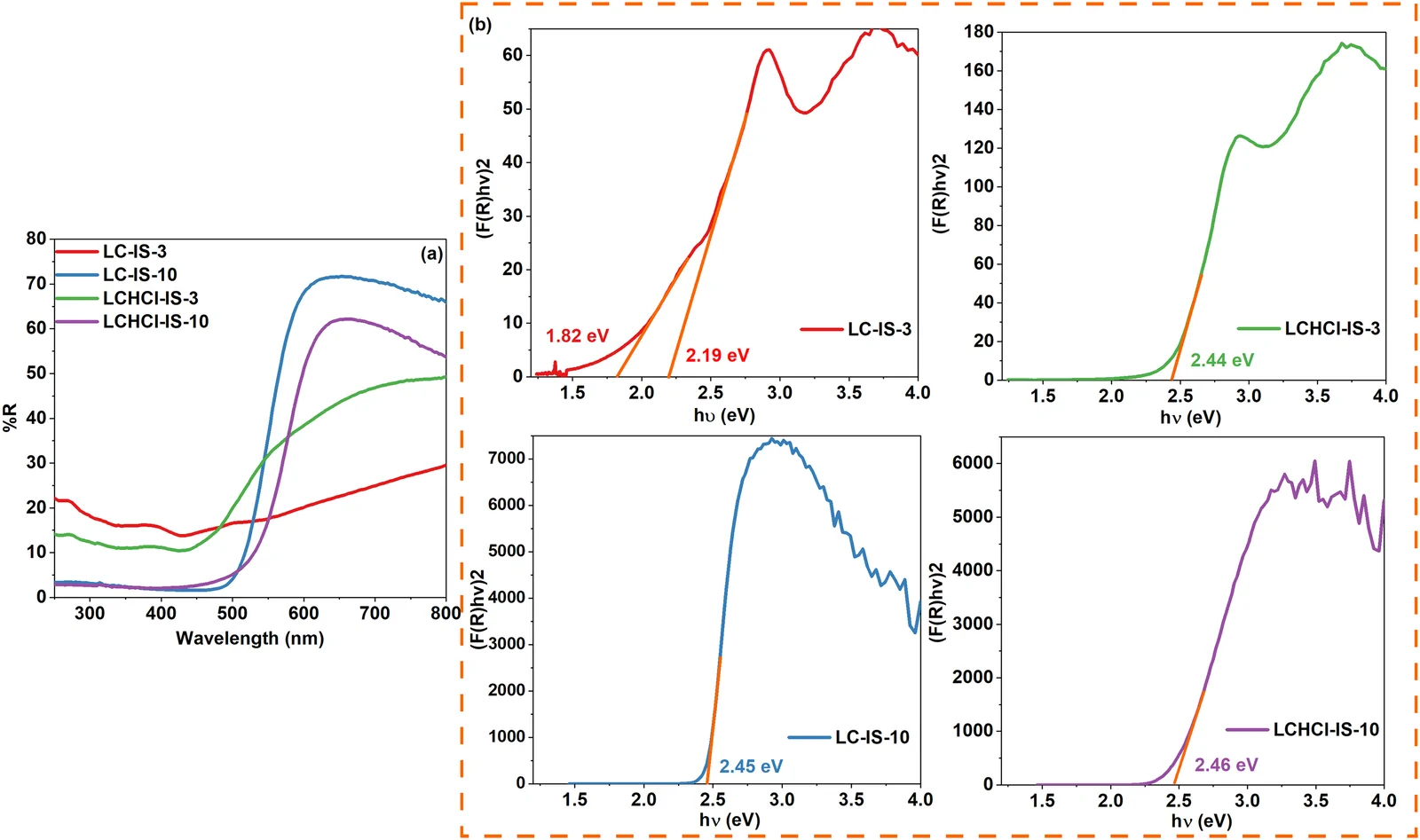
UV-vis DRS of the thin films (a) and (b) corresponding (*F*(*R*)*hv*)^2^*vs. hv* plots derived from the Kubelka–Munk function.^48^

The UV-vis diffused reflection spectra (DRS) are shown in Fig. 5. In Fig. 5a, the indium sulfide thin films LC-IS-3 and LCHCl-IS-3 have lower diffused reflectance intensities compared with the indium sulfide thin films LC-IS-10 and LCHCl-IS-10, of which, sample LC-IS-3 displays the lowest diffused reflection intensity possibly arising from its less defined edges and more fluid openings. While the more irregular, sponge-like structure in LCHCl-IS-3 might lead to greater light absorption than the vertically interconnected edged networks in sample LC-IS-10 and interconnected petal-like patterns in sample LCHCl-IS-10 by providing more absorbing sites. It is worth noting that LC-IS-10, with its vertically interconnected edges, has the highest diffused reflection intensity from 430 nm to 800 nm among all thin films. Moreover, the reflection edge of sample LC-IS-3 exhibits a significant red shift compared with other thin films, which could be associated with its unique surface chemistry, being more R–SH dominant than indium sulfide. The direct transition band gap energy values were determined from the spectra in Fig. 5b, derived from the Kubelka–Munk function.^48^ For thin film LC-IS-3, two distinct linear regions are observed in the magnitude plots of (*F*(*R*)*hv*)^2^*vs. hv*,^48^ attributed to the coexistence of In–cysteine complexes and indium sulfide phases.^49^ The higher-energy linear region corresponds to the direct band gap of the sample, estimated to be 2.19 eV; whilst the lower-energy region reflects an Urbach tail,^50^ associated with localized states induced by surface-capped In–cysteine complexes, yielding an indirect band gap of 1.82 eV. Accordingly, the direct band gaps for other thin films are determined to be 2.44 eV for LCHCl-IS-3, 2.45 eV for LC-IS-10, and 2.46 eV for LCHCl-IS-10. The variations in reflectance intensity and band gap suggest that the morphology and surface chemistry of the thin films play an important role in modulating their optical properties.^51,52^

**Fig. 6:**
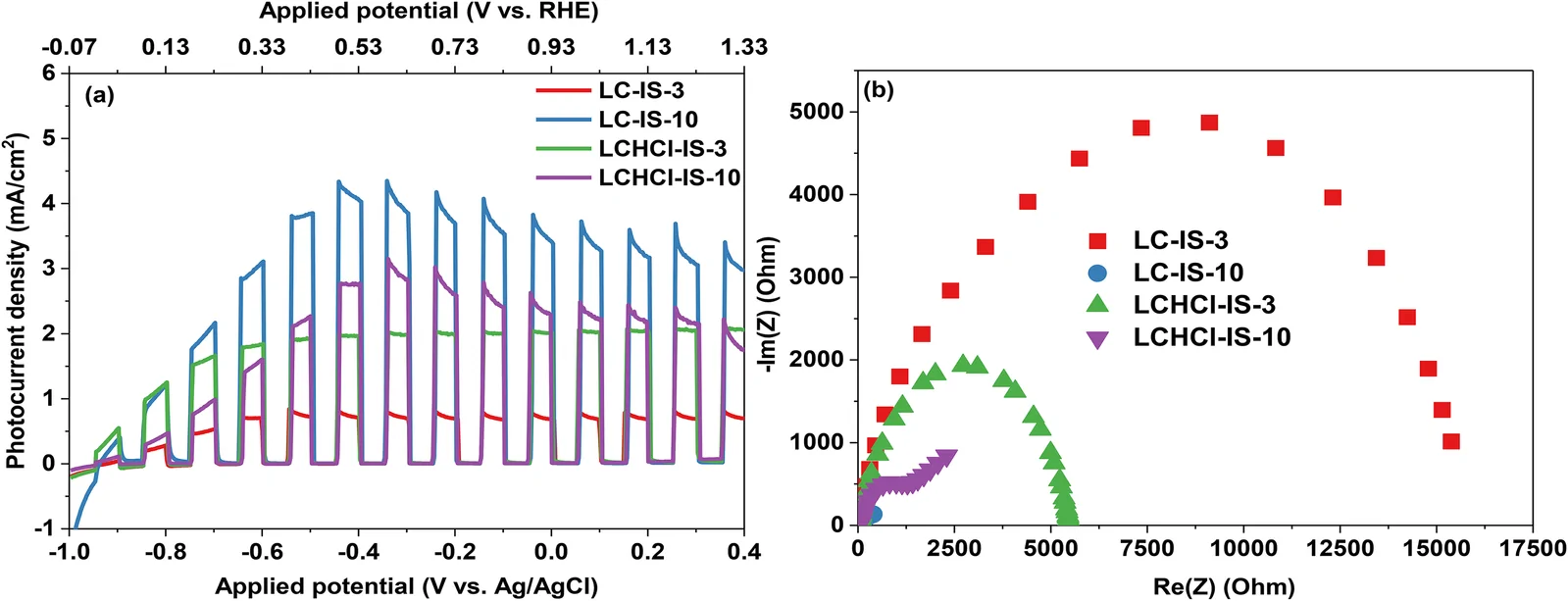
*J*–*V* plots under the chopped solar light irradiation (a) and (b) corresponding Nyquist plots recorded under constant solar light irradiation.

The *J*–*V* plots, along with the ESI results in Fig. 6 are presented to assess the PEC performance of the films. As illustrated in Fig. 6a, the thin films produced with a temperature ramp rate of 10 °C min^−1^ (LC-IS-10 and LCHCl-IS-10) exhibit higher photocurrent density than those generated with a temperature ramp rate of 3 °C min^−1^ (LC-IS-3 and LCHCl-IS-3). Specifically, at a bias of −0.2 V *vs.* Ag/AgCl, the LC-IS-10 thin film shows a photocurrent density of 3.7 mA cm^−2^, while the thin film LCHCl-IS-10 demonstrates a photocurrent density of 2.6 mA cm^−2^. In contrast, the thin film LCHCl-IS-3 exhibits a photocurrent density of 2.0 mA cm^−2^, and the thin film LC-IS-3 shows a markedly lower photocurrent density of 0.7 mA cm^−2^. Among these, the thin film LC-IS-10 notably outperforms previously reported In_2_S_3_-based photoelectrodes, as shown in Table S3. These variations can be attributed to the differences in film thickness caused by varying temperature ramp rates, with thicker indium sulfide thin films, especially those produced at lower temperature ramp rates, being less efficient for charge separation. Notably, the thin film synthesized using LC as the sulfur source demonstrates higher photocurrent density than that produced using LCHCl at the temperature ramp rate of 10 °C min^−1^, likely due to differences in morphology and changes in the dominant crystallographic facets. In contrast, the opposite trend is observed at the lower temperature ramp rate of 3 °C min^−1^, which is likely attributed to the predominance of the In–cysteine complexes in the LC-IS-3 thin film, which significantly hinders the separation efficiency of photogenerated charge carriers.

To comprehensively assess the PEC performance of the synthesized thin films, the applied bias photon-to-current efficiencies (ABPE) were calculated using the following eqn (2)^53–56^ and are presented in Fig. S5:2
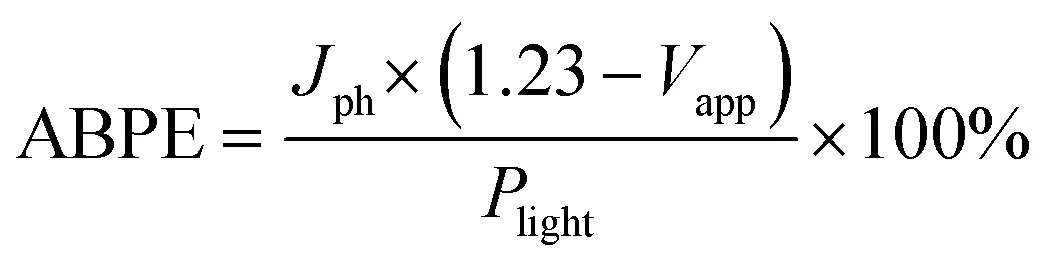
where *J*_ph_ is the photocurrent density (mA cm^−2^), *V*_app_ is the applied bias *vs.* RHE, and *P*_light_ is the incident light power (100 mW cm^−2^). At an applied potential of 0.73 V *vs.* RHE, the thin film LC-IS-10 exhibits the highest ABPE of 1.88%, followed by 1.30% for LCHCl-IS-10, 1.00% for LCHCl-IS-3, and 0.34% for LC-IS-3.

The Nyquist plots, as depicted in Fig. 6b, reveal that the thin film LC-IS-10 exhibits a small semicircle radius, indicating low charge transfer resistance at the interface between the thin film (or photoanode) and the electrolyte. In contrast, the thin film LC-IS-3 shows a larger semicircle radius, suggesting higher resistance.^57^ Thin film LCHCl-IS-10 and LCHCl-IS-3 display intermediate semicircle radii, indicating moderate charge transfer resistance levels.^57^

The Mott–Schottky analysis has been utilized to investigate the capacitance of the semiconductor–electrolyte junction under varying applied potentials. It provides valuable insights into the electronic properties of the semiconductor surface and the interfaces in contact with the electrolyte. The related effective carrier concentrations (*N*_eff_) of the indium sulfide thin films are determined by the following eqn (3):^58^3
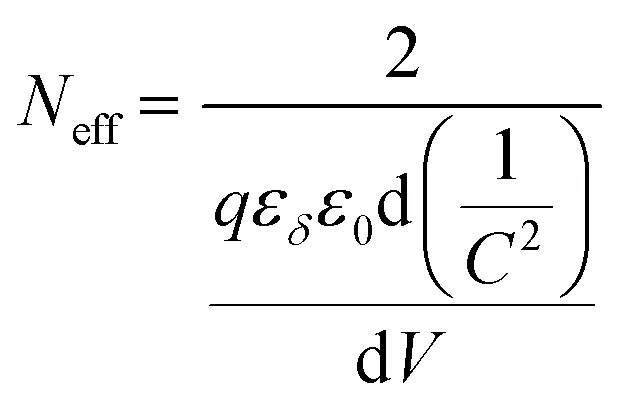
where *N*_eff_ is the effective carrier density, *q* is elemental charge constant with the value of (1.602 × 10^−19^ C),^59^*ε*_*δ*_ is the dielectric constant of In_2_S_3_ (8.5),^60–62^*ε*_0_ is the permittivity of vacuum (8.854 × 10^−14^ F cm^−1^),^61^ and 
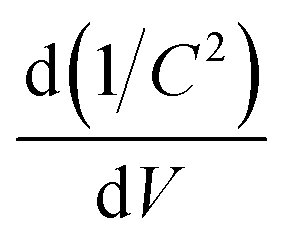
 represents the slope of the linear region in the Mott–Schottky plots.^59^ According to Fig. 8, all the slopes are positive, indicating a n-type semiconducting characteristic. This observation is consistent with the positive photocurrent shown in the *J*–*V* plots.^63^ Thin film LC-IS-3 exhibits a slope value of 3.9 × 10^11^, LCHCl-IS-3 shows 1.6 × 10^12^, LC-IS-10 has 4.1 × 10^10^, and LCHCl-IS-10 records 2.7 × 10^10^. Consequently, the carrier densities for LC-IS-3, LCHCl-IS-3, LC-IS-10, and LCHCl-IS-10 are estimated to be 4.25 × 10^17^ cm^−3^, 1.04 × 10^17^ cm^−3^, 4.03 × 10^18^ cm^−3^, and 6.22 × 10^18^ cm^−3^, respectively. The flat band potential (*V*_fb_) of an n-type semiconductor is typically 0.1–0.2 V more positive than its conduction band edge potential (*V*_CB_).^64^ In this study, a 0.1 V offset is used to estimate *V*_CB_ from the Mott–Schottky-derived *V*_fb_. For thin films LC-IS-3, LCHCl-IS-3, LC-IS-10, and LCHCl-IS-10, the *V*_fb_ values are estimated to be −0.75 V, −0.86 V, −0.83 V, and −0.80 V *vs.* Ag/AgCl, respectively. These values correspond to −0.17 V, −0.28 V, −0.25 V, and −0.22 V *vs.* RHE, respectively. Accordingly, *V*_CB_ values are estimated at −0.27 V, −0.38 V, −0.35 V, and −0.32 V *vs.* RHE, respectively. Based on the band gap values, the valence band potentials (*V*_VB_) of the indium sulfide thin films are calculated using the following eqn (4):^65^

4 *E*_c_ = *E*_v_ − *E*_g_

**Fig. 7:**
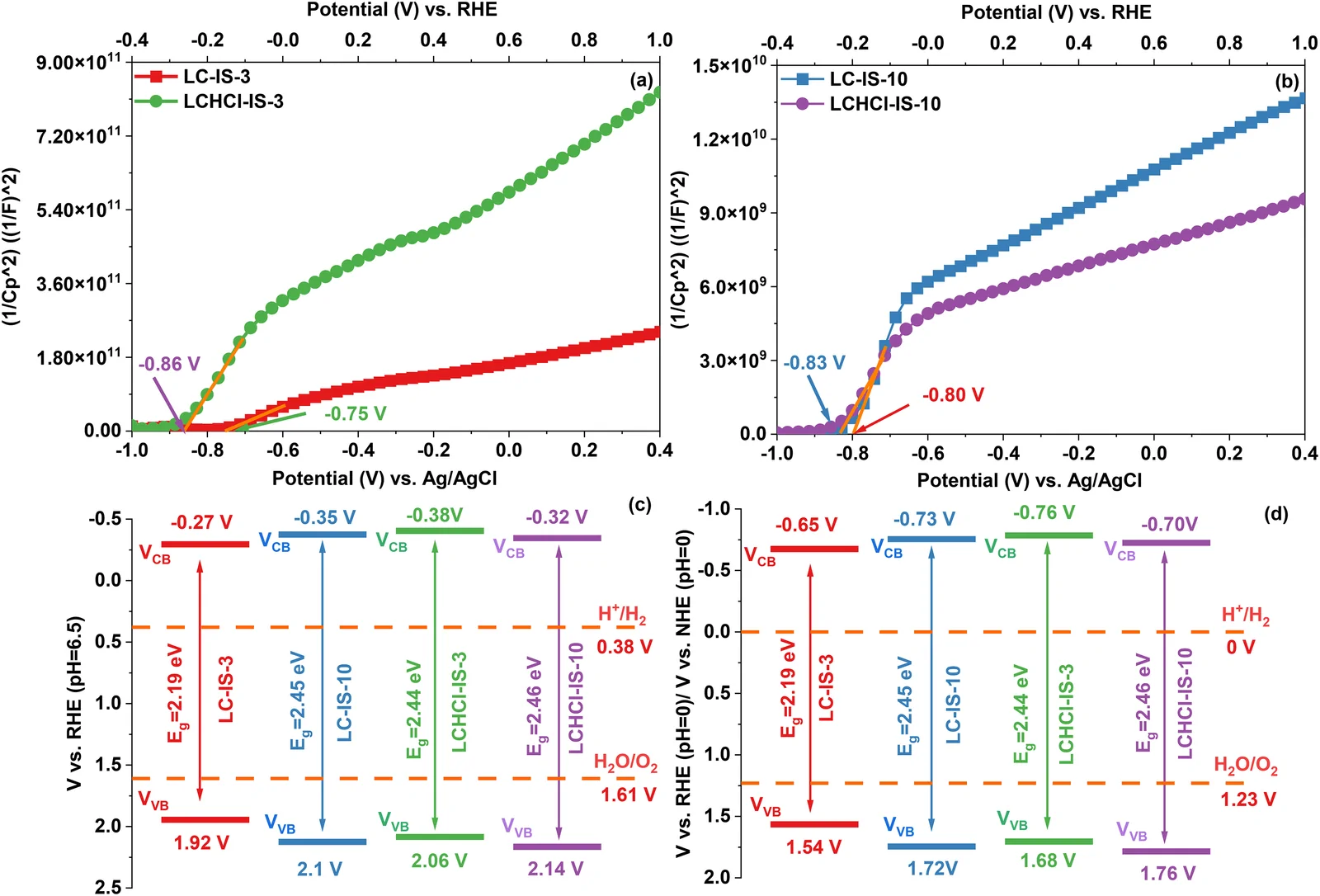
Mott–Schottky plots of the prepared indium thin films were generated in 0.5 M Na_2_SO_4_ (pH = 6.5) (a) and (b); band edge potentials *vs.* RHE at pH 6.5 (c); band edge potentials *vs.* RHE or NHE at pH 0 (d).

Finally, the electronic band edge potential positions are determined and depicted in Fig. 7c for pH 6.5, with the corresponding band edge potential alignment at pH 0 calculated using the McAuley group's calculator^66^ and being illustrated in Fig. 7d. All the thin films exhibit suitable band edge alignment, with conduction band potentials being more negative than the water reduction potential (0.38 V *vs.* RHE at pH 6.5/0 V *vs.* NHE at pH 0) and valence band potentials being more positive than the water oxidation potential (1.61 V *vs.* RHE at pH 6.5/1.23 V *vs.* NHE at pH 0).^67^

**Fig. 8:**
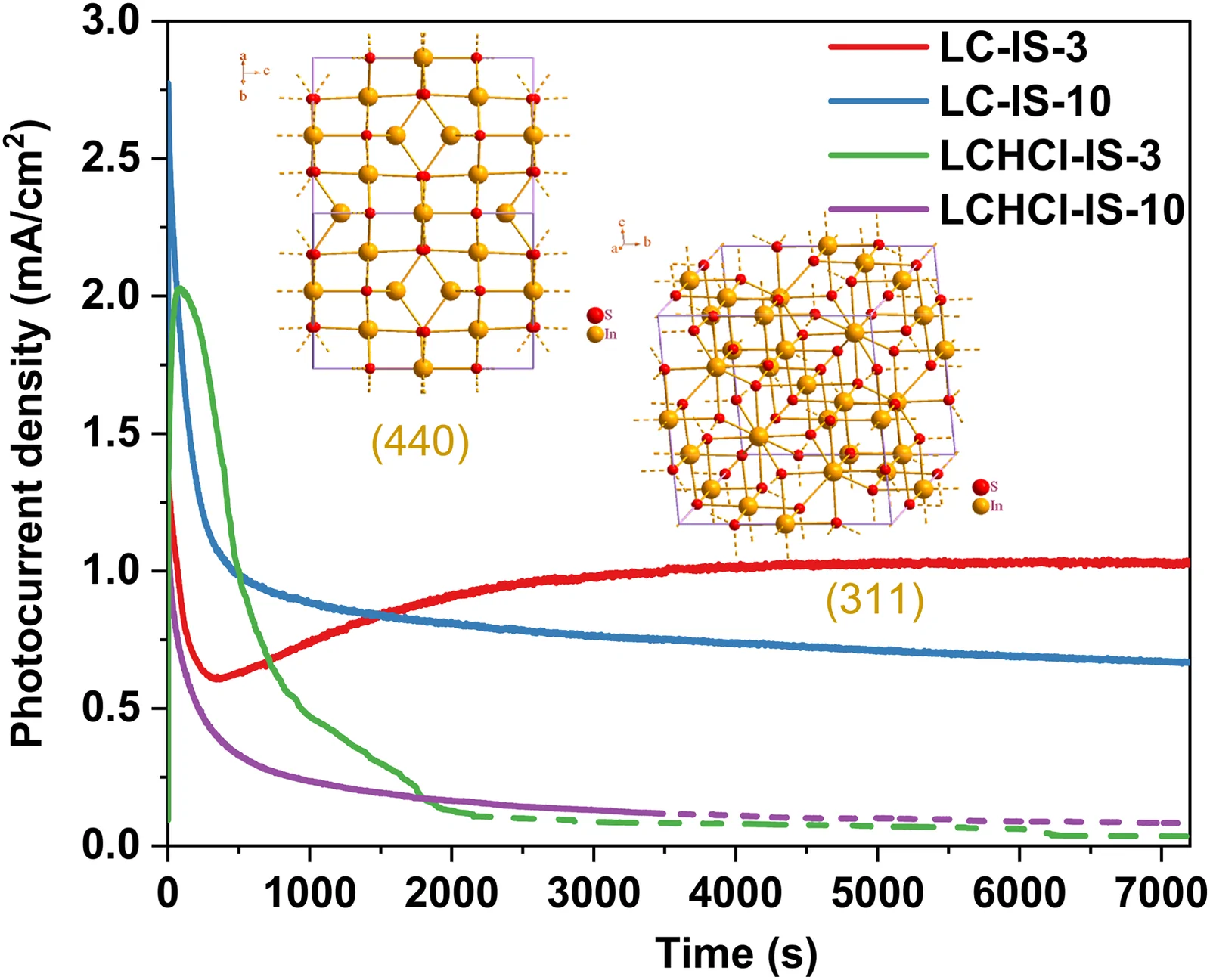
Long-term PEC water splitting stability of indium sulfide thin films under continuous 1 sun illumination using a mixture of 0.025 M Na_2_S·9H_2_O and 0.025 M Na_2_SO_3_ electrolyte solution (pH = 12.32). The inset highlights the strucutre of the crystallographic facets (311) and (440).

The photocurrent stability was evaluated using an external bias of −0.2 V *vs.* Ag/AgCl under continuous 1 sun illumination. As illustrated in Fig. 8, a drastic drop in the photocurrent density occurred within 500 seconds of illumination, attributed to the photocorrosion of both In–cysteine complexes and indium sulfide.^68,69^ This degradation could be ascribed to the oxidation of the S^2−^ species, which leads to the breakdown of In–cysteine complexes and partial disruption of the indium sulfide crystal structure. Under continuous illumination, the photocurrent densities of thin films LC-IS-3 and LC-IS-10 remained stable at 1 mA cm^−2^ and 670 μA cm^−2^, respectively, surpassing the values reported by Chen's group (500 μA cm^−2^)^70^ and Wang's group (20 μA cm^−2^).^8^ In contrast, the photocurrent densities of the films LCHCl-IS-3 and LCHCl-IS-10 exhibited a continuous decline, dropping to 33 μA cm^−2^ and 90 μA cm^−2^, respectively, after 2 hours of illumination. These results indicated that thin films prepared using LC had higher stability than those prepared with LCHCl. A summary of the key structural and PEC properties, including crystallographic orientation, film thickness, photocurrent density, and stability for all thin films, is provided in Table 3 to facilitate comparison. This difference could be attributed to the predominant exposure of the (440) facet in LC-prepared thin films, whereas LCHCl-prepared films primarily exposed the (311) facet. According to the crystal figures of the (440) and (311) facets shown in Fig. 8, the (440) facet has more densely packed and symmetric atomic arrangement, along with reduced sulfur exposure, compared with the (311) facet, which is more open, structurally complex, and characterized by irregular spacing and lower atomic packing density. The reduced sulfur exposure contributed to the stability of the films, even after undergoing photocorrosion caused by the oxidation of S^2−^ species.^69^ In contrast, the high sulfur exposure of the (311) facet led to the destruction of the crystal structure during photocorrosion, causing the films to peel off and resulting in lower long-term photocurrent stability. Notably, a subsequent increase in photocurrent density after the initial sharp drop was observed for sample LC-IS-3. According to the S 2p XPS results, the surface of thin film LC-IS-3 is dominated by indium–cysteine complexes, whereas LC-IS-10 primarily consists of indium sulfide capped by a smaller amount of these complexes. Additionally, the difference in temperature ramp rates during synthesis results in LC-IS-3 having thicker film (6.2 μm) compared with LC-IS-10 (3.5 μm). This increased thickness initially leads to higher charge combination and transport limitations, causing a lower photocurrent in LC-IS-3. However, under illumination, the indium–cysteine complexes are unstable and undergo photocorrosion. Their degradation later contributed to changes in film thickness and improved charge transport. The photocurrent density of sample LCHCl-IS-3 exhibited a slower decline, likely due to the sacrificial role of surface-capped In–cysteine complexes, which shielded the (311) facet from photocorrosion, thereby mitigating the decrease in photocurrent density. However, its greater thickness compared with LCHCl-IS-10 and the oxidation of surface S^2−^ species eventually caused the film to peel off, disrupting interfacial charge transfer and leading to PEC water-splitting ability losses. However, the exfoliated film may still retain intrinsic PEC property.

**Table 3:** Comparison of structural and PEC properties

Thin film	Crystallographic orientation	Film thickness (μm)	Photocurrent density (mA cm^−2^ at −0.2 V *vs.* Ag/AgCl)	Stability (mA cm^−2^)
LC-IS-3	(440)	6.2	0.7	1
LC-IS-10	(440)	3.5	3.7	0.67
LCHCl-IS-3	(311)	9.2	2	0.033
LCHCl-IS-10	(311)	2.7	2.6	0.090

## Conclusions

The utilization of LC and LCHCl as sulfur precursors in the hydrothermal synthesis of indium sulfide thin films, with controlled temperature ramp rates, had been successfully demonstrated. The selection of sulfur precursors and the variation in temperature ramp rates significantly influenced the crystallographic orientation, morphology, thickness, electronic properties, and the PEC water splitting performance of the indium sulfide thin films. Specifically, LC favoured the formation of the (440) facet, while LCHCl promoted the (311) facet, resulting in distinct morphologies. Controlling the temperature ramp rates also significantly affected the crystallite sizes and thickness of the indium sulfide thin films. Lower temperature ramp rate led to thicker films with larger crystallite sizes, which significantly impacted the charge separation efficiency and resulted in reduced PEC water splitting performance. Moreover, the LC-synthesized indium sulfide thin films, dominated by the (440) plane, exhibited a more compact and symmetric atomic arrangement with reduced sulfur exposure, which likely contributed to their superior long-term photocurrent stability compared with those enriched with (311) facet, which features a more open structure and sulfur exposure. These fundings highlight the importance of sulfur precursor selection and synthesis parameters in optimizing the crystallographic orientation, morphology, and overall PEC water splitting performance of indium sulfide thin films, providing valuable insights for advancing the design of materials for PEC water splitting applications.

## Conflicts of interest

The authors declare that they have no known competing financial interests or personal relationships that could have appeared to influence the work reported in this paper.

## Supplementary Material

LF-002-D5LF00219B-s001

## Data Availability

Supplementary information: The SI includes EDS and XPS spectra, ABPE plots, and tables summarizing atomic ratios, XRD facet intensity ratios, and reported PEC performance of In_2_S_3_ photoelectrodes. See DOI: https://doi.org/10.1039/D5LF00219B. All data supporting the findings of this study are included within the article and its SI.
