# Supplementary material for: Tailoring the crystallographic orientation and thickness of indium sulfide thin films for enhanced photoelectrochemical water splitting
Source: RSC Appl Interfaces. 2025 Sep 3;2(6):1734–46. doi: 10.1039/d5lf00219b (PMC13591375; doi:10.1039/d5lf00219b)
Supplement: Supplementary file 1 [file LF-002-D5LF00219B-s001.pdf]

Supplementary Information (SI) for RSC Applied Interfaces.
This journal is © The Royal Society of Chemistry 2025

Supporting Information

Tailoring the Crystallographic Orientation and Thickness of Indium Sulfide Thin Films for Enhanced Photoelectrochemical Water Splitting

Xiuru Yang[a], Arthur Graf[b, c], Hong Chang[a], Yongde Xia[a], Asif Ali Tahir*[d], Yanqiu Zhu*[a]

[a] Faculty of Environment, Science and Economy, University of Exeter, Exeter EX4 4QF, UK

[b] HarwellXPS, Research Complex at Harwell, Rutherford Appleton Lab, Didcot, OX11 0FA UK

[c] School of Chemistry, Cardiff University, Main Building, Park Place, Cardiff, CF10 3AT UK

[d] Solar Energy Research Group, Environment and Sustainability Institute, Faculty of Environment, Science and Economy, University of Exeter, Penryn Campus, Penryn TR10 9FE, UK

*Corresponding authors

E-mail: y.zhu@exeter.ac.uk

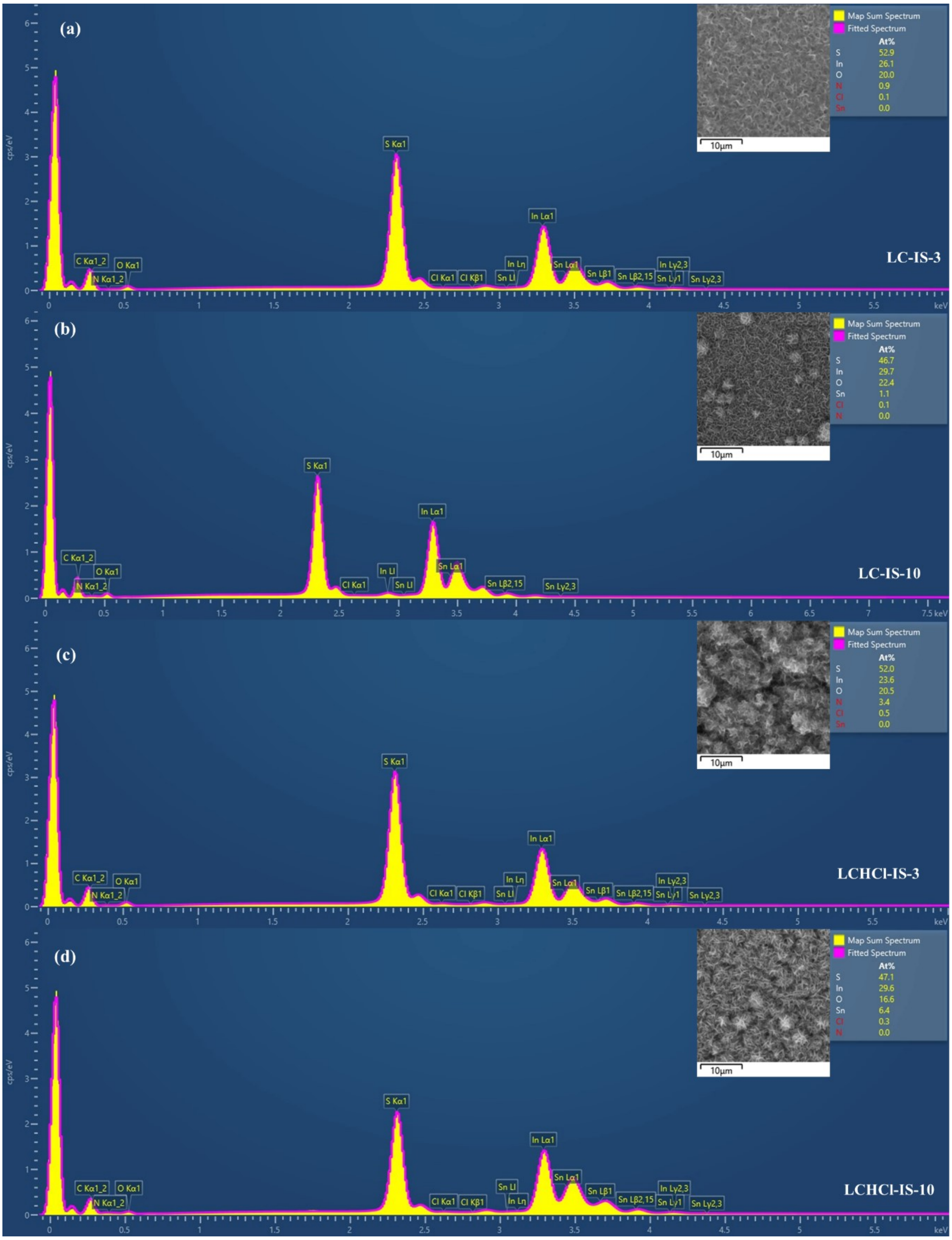

Figure S1. EDS spectra of the prepared indium sulfide thin films: (a) LC-IS-3, (b) LC-IS-10, (c) LCHCl-IS-3, and (d) LCHCl-IS-10.

Table S1 Atomic ratio (At%) of the prepared thin films

Thin films \ Atomic ratio (At%)	In	S	O	N	Cl	Sn	In: S
**LC-IS-3**	26.1	52.9	20.0	0.9	0.1	0	1: 2
**LC-IS-10**	29.7	46.7	22.4	0	0.1	1.1	1: 1.6
**LCHCl-IS-3**	23.6	52	20.5	3.4	0.5	0	1: 2.2
**LCHCl-IS-10**	29.6	47.1	16.6	0	0.3	6.4	1: 1.6

Table S2 Intensity Ratios of Dominant Crystallographic Facets from Thin Film XRD Pattern Analysis.

Thin Film	$I_{(311)}$	$I_{(440)}$	$I_{(311)}/I_{(440)}$
**LC-IS-3**	216	576	0.38
**LC-IS-10**	113	147	0.77
**LCHCl-IS-3**	946	578	1.64
**LCHCl-IS-10**	500	253	1.98

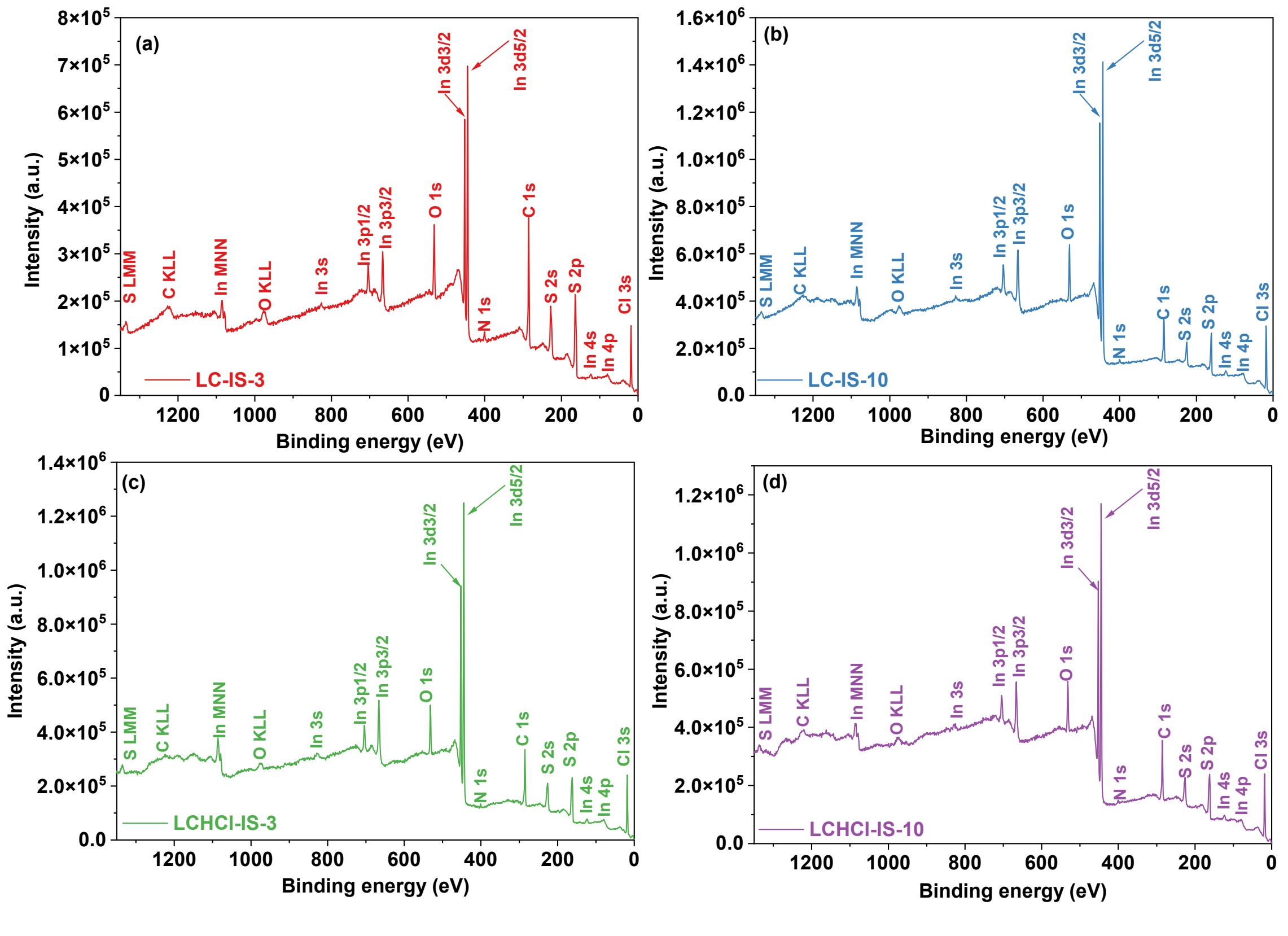

Figure S2. The XPS survey scan spectra of prepared indium sulfide thin films: (a) LC-IS-3, (b) LC-IS-10, (c) LCHCl-IS-3, and (d) LCHCl-IS-10.

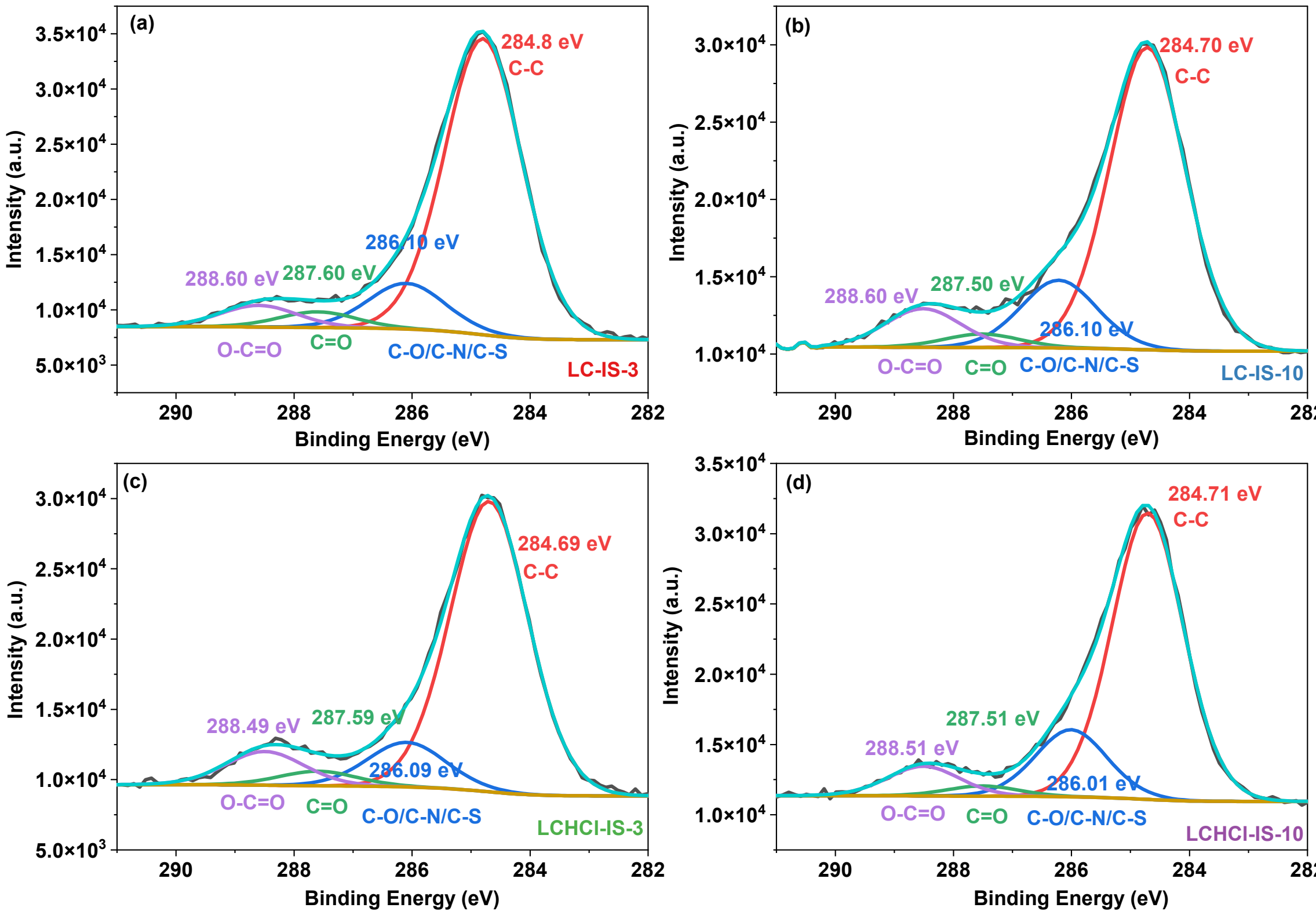

Figure S3. C 1s spectra of prepared thin films: (a) LC-IS-3, (b) LC-IS-10, (c) LCHCl-IS-3, and (d) LCHCl-IS-10.

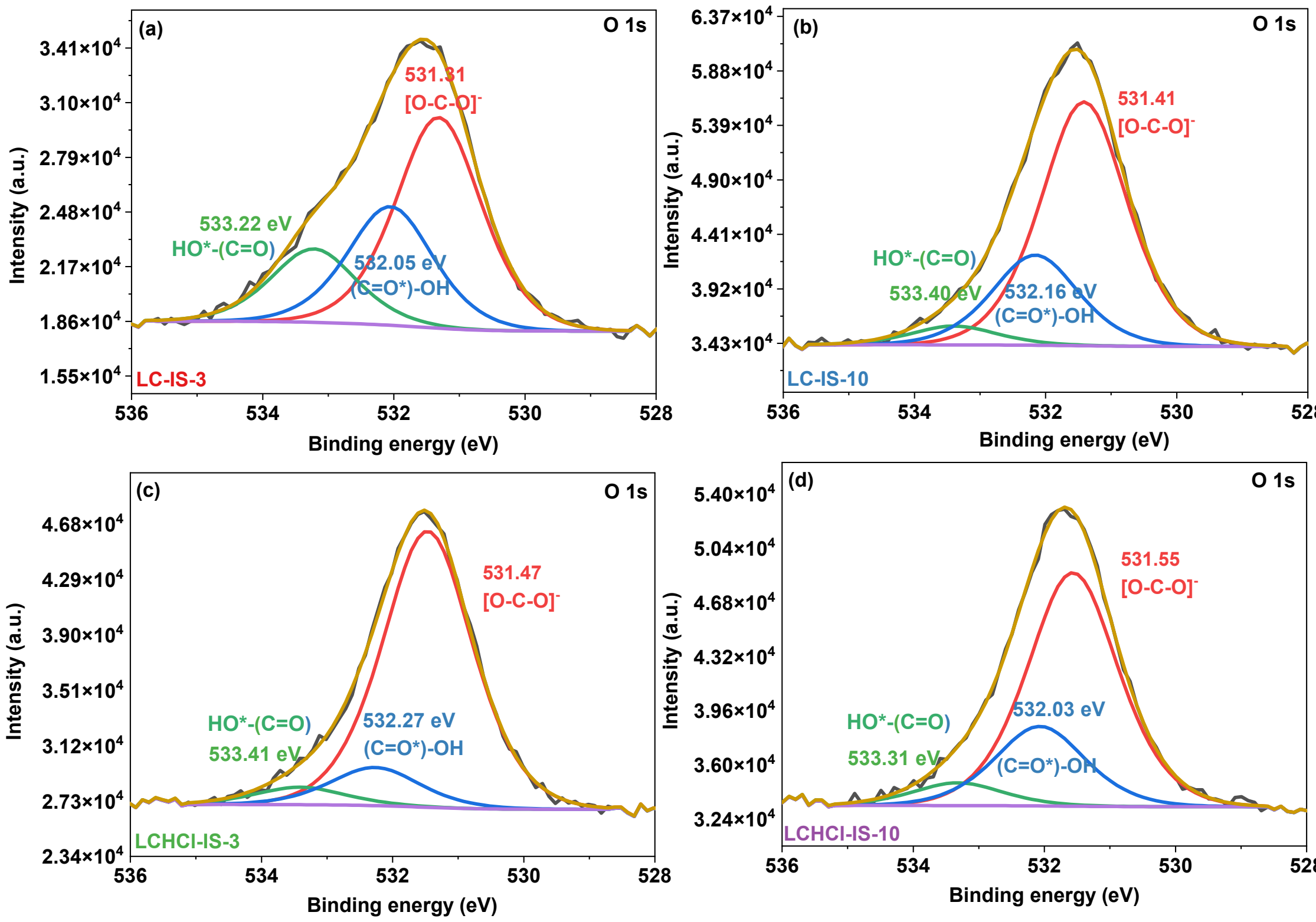

Figure S4. O 1s spectra of prepared thin films: (a) LC-IS-3, (b) LC-IS-10, (c) LCHCl-IS-3, and (d) LCHCl-IS-10.

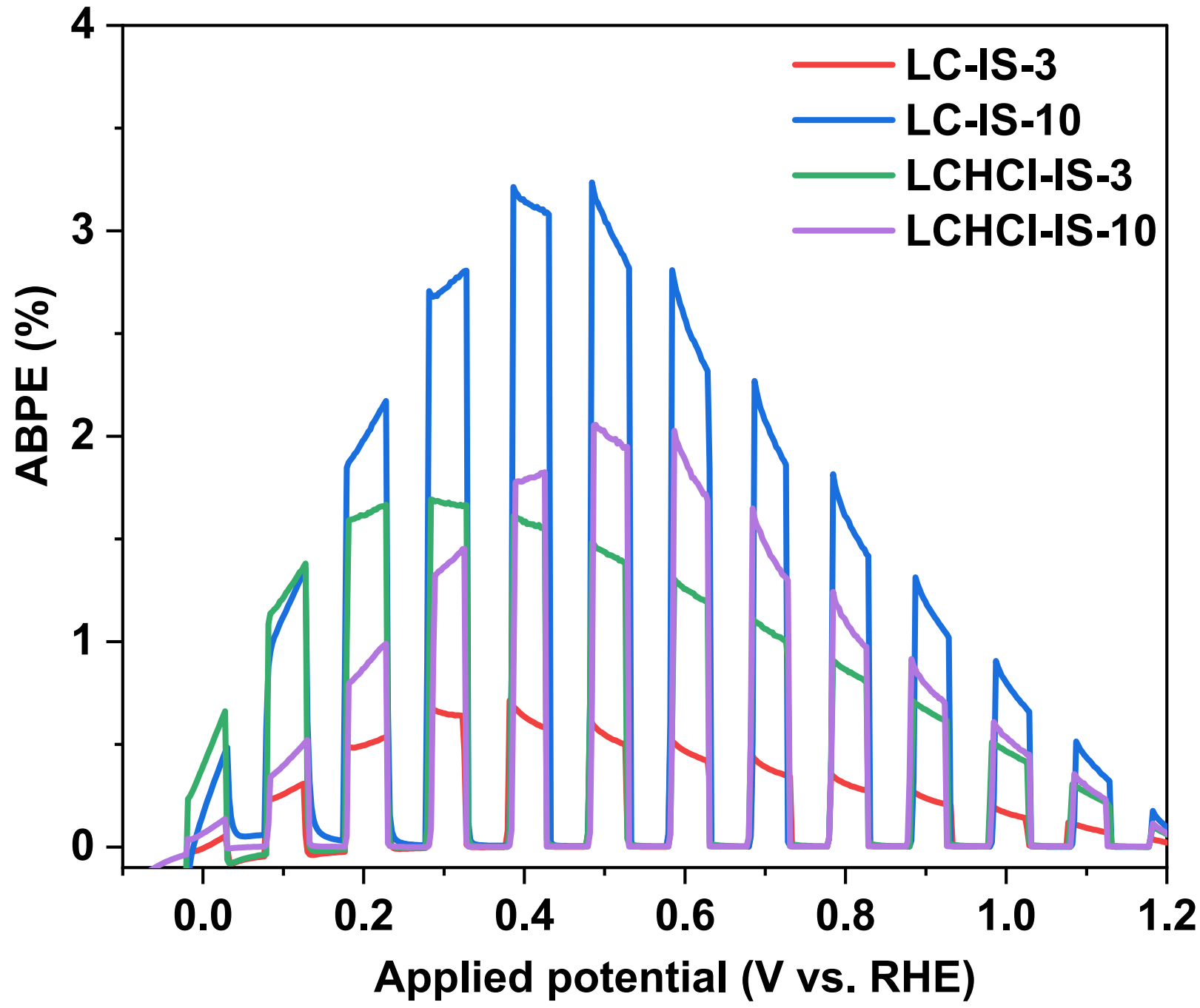

Figure S5. Applied bias photo-to-current efficiency (ABPE) plots derived from LSV plots under chopped AM 1.5 G illumination (100 mW/cm^2).

Table S3 Representative In_2S_3 photoelectrodes reported in the literature and their corresponding PEC performance.

	Synthesis method	electrolyte	Pt counter (shape or size)	PEC cell atmosphere	J (mA/cm^2) (applied potential)	J (mA/cm^2) (applied time)	Ref.
In_2S_3	hydrothermal	0.35 M Na_2SO_3 and 0.25 M Na_2S	Pt (foil)	N/A	2.7 (0.78 V vs. RHE)	2 (2400 seconds)	1
In_2S_3	solvothermal	0.10 M $Na_2S_2O_3$	Pt (sheet)	N/A	1.3 (0.2 V vs. Ag/AgCl)	N/A	2
In_2S_3	solvothermal	1.0 M KCl	Pt mesh	N/A	0.0357 (1.2 V vs. RHE)	N/A	3
In_2S_3	hydrothermal	0.25 M Na_2SO_4	N/A	N/A	1 (0.5 V vs. Ag/AgCl)	0.8 (300 seconds)	4
β-In_2S_3	spin-coating	0.5 M NaOH	Pt mesh (0.8 mm^2)	purged with N_2	0.1(1.3 V vs. RHE)	0.01 (240 min)	5
In_2S_3	aerosol-assisted chemical vapour deposition (AACVD)	0.01 M Na_2SO_4 and 0.10 M Na_2S	Pt (N/A)	N/A	1.25 (0.23 V vs. Ag/AgCl)	N/A	6

References

1. X. Feng, Y. Chen, M. Wang and L. Guo, *Int. J. Hydrogen Energy*, 2017, **42**, 15085-15095, https://doi.org/10.1016/j.ijhydene.2017.04.283.

2. F.-Y. Su, W.-D. Zhang, Y.-Y. Liu, R.-H. Huang and Y.-X. Yu, *J. Solid State Electrochem.*, 2015, **19**, 2321-2330, https://doi.org/10.1007/s10008-015-2868-x.

3. M. Li, X. Tu, Y. Su, J. Lu, J. Hu, B. Cai, Z. Zhou, Z. Yang and Y. Zhang, *Nanoscale*, 2018, **10**, 1153-1161, https://doi.org/10.1039/c7nr06182j.

4. F. Jrad, J. B. Naceur, R. Ouertani and R. Chtourou, *Phys. E Low-dimens. Syst. Nanostruct.*, 2019, **114**, 113585, https://doi.org/10.1016/j.physe.2019.113585.

5. L. Wang, L. Xia, Y. Wu and Y. Tian, *ACS Sustain. Chem. Eng.*, 2016, **4**, 2606-2614, https://doi.org/10.1021/acssuschemeng.6b00090.

6. M. A. Ehsan, T. A. Peiris, K. G. Wijayantha, M. M. Olmstead, Z. Arifin, M. Mazhar, K. M. Lo and V. McKee, *Dalton Trans.*, 2013, **42**, 10919-10928, https://doi.org/10.1039/c3dt50781e.
